# Using zeta potential to study the ionisation behaviour of polymers employed in modified-release dosage forms and estimating their pK_a_

**DOI:** 10.1016/j.ijpx.2019.100024

**Published:** 2019-07-19

**Authors:** Joao A.C. Barbosa, Malaz S.E. Abdelsadig, Barbara R. Conway, Hamid A. Merchant

**Affiliations:** Department of Pharmacy, School of Applied Sciences, University of Huddersfield, Queensgate, Huddersfield HD1 3DH, United Kingdom

**Keywords:** pK_a_, Ionisation, Enteric, Gastro-resistant, Modified-release, Zeta-potential, Charge, Dissolution

## Abstract

A range of enteric polymers is used in pharmaceutical industry for developing gastro-resistant formulations. It is generally implied that these coatings are interchangeable due to similar dissolution pH thresholds reported by suppliers. Despite rapid dissolution in compendial phosphate buffers, these products can take up to 2 h to disintegrate *in-vivo* in the human small intestine. The factors primarily responsible for such variability in dissolution of these polymeric coatings are the differences in ionisation of acidic functional groups on polymer chains and their interplay with ions and buffer species present in gastrointestinal fluids. In this study, we aim to develop a novel, simple and inexpensive technique that can be used under various *in-vitro* conditions to study the ionisation behaviour of commonly used polymers (EUDRAGIT-E100, L100, S100, HPMC AS-LF, AS-HF, HP-50, HP-55) and to estimate their pK_a_. Moreover, this method was successfully applied to study the ionisation behaviour of a range of natural polymers (Guar, Tara, locust bean, Konjac gums, gum Arabic, citrus pectin, chitosan and alginate) and their pK_a_ was also estimated. The proposed method would allow a better understanding of the dissolution behaviour of these polymers within gastrointestinal tract and will aid rational design of modified release dosage forms.

## Graphical abstract

**Figure:**
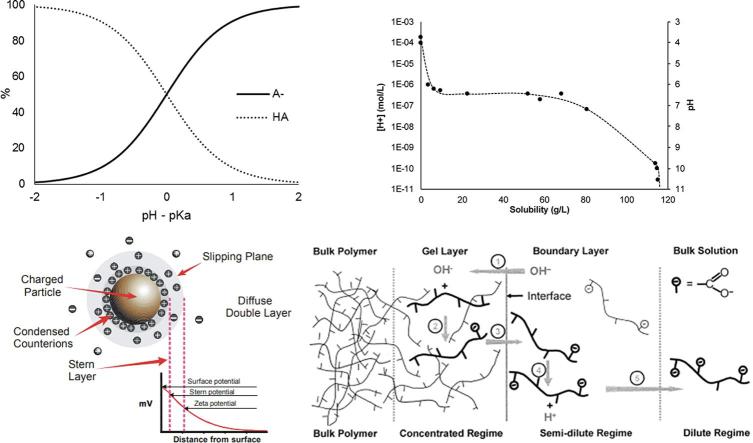

## 1 Introduction

**Fig. 1:**
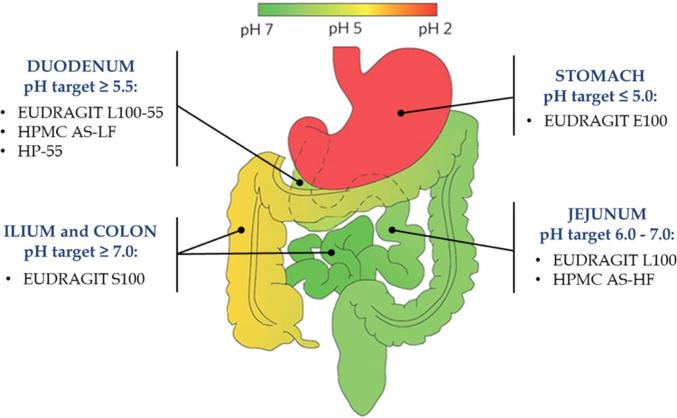
Schematic showing different polymers used to target drugs in the human gastrointestinal tract; Adapted from (Khutoryanskiy, 2015).

Different types of enteric polymers are used in pharmaceutical industry to develop delayed-release formulations targeting different parts of the gastrointestinal (GI) tract (Fig. 1). It is generally implied, due to their similar dissolution pH thresholds reported by suppliers, that these materials are interchangeable provided the drug release from these products in conventional buffers is similar.

Despite rapid dissolution in compendial phosphate buffers, most gastro-resistant products designed to release drug in proximal small intestine, can take up to 2 h to disintegrate after emptying from stomach (Varum, 2014), which markedly demonstrates the underperformance of the compendial *in-vitro* test method to predict *in-vivo* behaviour of these formulations. However, it has been reported that in physiologically relevant buffers, remarkable differences in dissolution profiles were observed between various polymer-coated tablets, which is in agreement with the delayed disintegration times reported in the literature (Liu, 2011). Therefore, the pH dependent dissolution of these polymers generally depends on their ionisation behaviour in the luminal environment within GI tract, and in-depth understanding can therefore provide invaluable insights to understand how these polymeric materials behave in different pH conditions within the GI tract.

**Fig. 2:**
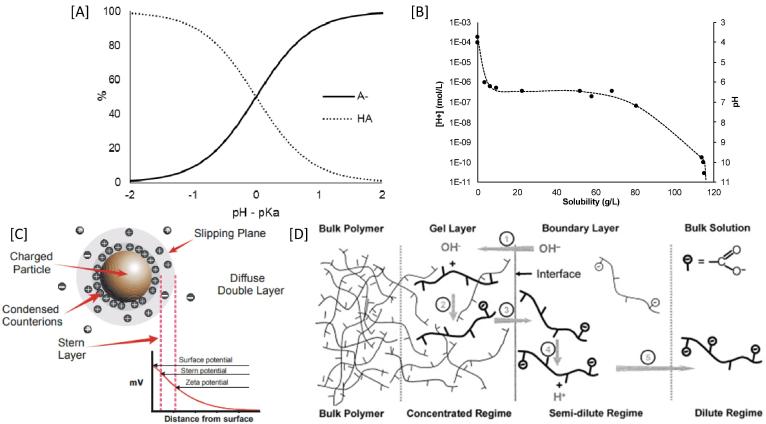
[A] pH-dependant ionisation of a weak acid [HA] and its conjugated base [A^-^] drawn using Henderson-Hasselbalch equation; [B] Ionisation and solubility of a pH-responsive polymer as a function of pH (redrawn using data from (Nguyen and Fogler, 2005); [C] A schematic showing the potential difference as a function of distance from the charged surface of a particle in a medium (Malvern, 2017); [D] Dissolution mechanism of pH-responsive polymers reproduced with permission from (Nguyen and Fogler, 2005). The encircled numbers in [D] represent (1) Diffusion of water and hydroxyl ions into the polymer matrix to form a gel layer, (2) Ionization of polymer chains in the gel layer, (3) Disentanglement of polymer chains out of the gel layer to the polymer-solution interface, (4) Further ionization of polymer chains at the polymer interface, (5) Diffusion of disentangled polymer chains away from the interface toward the bulk solution.

*In-vivo* dissolution of these polymeric coatings is a complex interplay between the ionisation constant of the polymer and the characteristics of gastrointestinal fluid, such as fluid volume, ionic concentration, buffer species, their pK_a_ and capacity. According to the Henderson-Hasselbalch equation, the pK_a_ of a weak acid corresponds to the environmental pH at which the concentration of the weak acid ([HA]) equals the concentration of its conjugated base ([A^−^]). At this pH, the weak acid will tend to partially ionise; whereas almost a full ionisation is expected when the environmental pH is 2-units above its pK_a_ (Fig. 2A).

There have been several techniques reported in the literature to determine pK_a_ value of acids, for instance: UV–vis spectrometry, conductometry, solubility, electrophoresis, partition coefficients, NMR, polarimetry, voltammetry, HPLC, fluorometry, calorimetry, surface tension and computational (Dickhaus and Priefer, 2015); many techniques work well for small molecules but there are limitations on measurements involving large polymeric species. The use of zeta potential has been previously described to study the acid-base equilibria of multi-layered weak polyelectrolytes assembled on silica particles (Burke and Barrett, 2003, Mateo and Priefer, 2015). The enteric polymers employed as gastroresistant coatings behave as weak acids in solution and exhibit a pH-dependant ionisation. At a pH above the polymer’s pK_a_ value, the carboxylic acid groups tend to ionise, increasing [A^−^] and proportionally decreasing [HA] (Fig. 2A), which will increase polymer’s solubility, leading to complete dissolution (Fig. 2B). The ionisation of an acidic polymer produces a proportional increase in negatively charged groups yielding a net negative charge on the polymer. The charging behaviour at this solid-liquid interface can be described by the zeta potential as it represents the electrical potential at the shear plane, which separates a stationary layer and a mobile layer of charges (Fig. 2C). Thus, increasing ionisation results in a proportional increase in the zeta potential of a polymeric material suspended in a medium. The maximum absolute value of the zeta potential (Zeta_max_), therefore corresponds to the maximum ionisation of the polymer, i.e. when [A^−^] is maximal. Hence, an equal concentration of the weak acid to the concentration of its conjugated base ([A^−^] = [HA]) can be attributed to the half of the Zeta_max_, and the corresponding pH of the medium will correspond to the pK_a_ value of the polymer.

In this study, we aimed to develop a simple and economical technique of determining pK_a_ values of various polymeric materials, which can be adopted in different *in-vitro* conditions to study their ionisation behaviour in a range of pharmaceutical applications, in particular with modified-release dosage forms.

## 2 Materials and methods

### 2.1 Materials

The acrylic (EUDRAGIT®) and cellulose based (HPMC AS/P) enteric polymers used in this study were provided as samples from Evonik Industries AG, Germany and Shin-Etsu, Japan, respectively and their properties are summarised in Table 1. Hydrochloric acid NIST 1M and sodium hydroxide NIST 1M solutions were purchased from Fisher Scientific (Leicestershire, UK). Tara and Konjac gums were obtained from Ingredients UK Limited (Hampshire, UK). Citrus pectin (P9135), guar gum (G4129), gum Arabic, chitosan (75–85% deacetylation, 448877) and κ-carrageenan (22048) were purchased from Sigma-Aldrich (Dorset, UK). Locust bean gum (GC1233) was purchased from Glentham Life Sciences (Wiltshire, UK). Supplier product codes for the natural gums are given in brackets.

**Table 1:** Synthetic polymers used in this study and their characteristics.

Polymer	Product name	Grade	Dissolution pH threshold	% ionisable groups	M.W. (g/mol)	Manufacturer/supplier
Methacrylic acid copolymer	EUDRAGIT®	Dimethyl amino ethyl^[1]^				Evonik GmbH, Darmstadt, Germany
		E100	≤5.0	20.8–25.5	47,000	
		Methacrylic acid^[2]^				
		L100	≥6.0	46.0–50.6	125,000	
		S100	≥7.0	27.6–30.7	125,000	

HPMC acetate succinate (AS)	Aqoat®	Succinoyl^[3]^				Shin-Etsu Chemical Co., Ltd., Japan
		LF	≥5.5	14.0–18.0	18,000	
		HF	≥6.8	4.0–8.0	18,000	
HPMC phthalate (HP)	HPMCP	Phthalyl^[4]^				
		HP-50	≥5.0	21.0–27.0	78,000	
		HP-55	≥5.5	27.0–35.0	84,000	

[1]: Evonik, 2015; [2]:Evonik, 2012; [3]: Shin-Etsu, 2005; [4]: Shin-Etsu, 2002.

### 2.2 Preparation of polymeric dispersions

Polymeric suspensions were prepared in 0.1 M HCl at different concentrations (0.1–0.5% w/v). When necessary, a homogenizer was used (Silverson L5M) in order to assure adequate dispersion. Polymer concentrations, mixing and homogenization times were optimised to produce a homogenous dispersion of the polymers at low pH.

### 2.3 Method validation for pK_a_ determination

To evaluate the validity of using zeta potential measurements in determining the pK_a_ value of different polymers, a selection of commonly used and well-known synthetic enteric-polymers was used. The polymers characteristics are summarised in Table 1. Upon validation, the method was then employed to study the ionisation behaviour of various natural polymeric materials (Table 2).

**Table 2:** Natural polymers used in this study and their food and pharmaceutical applications.

Gum	Structure	Common uses and applications
1. *Gums containing acidic moieties*		
Gum Arabic	Main chain consisting of β-(1,3) linked galactose units with branches of β-(1,6) linked galactose and arabinose with terminal rhamnose and glucuronic acid. Contains 2% of protein within the structure^[1]^.	Suspending agent, emulsifying agent, binder in tablets, demulcent and emollient in cosmetics^[2,3]^, osmotic drug delivery^[4]^.
Pectin	Linear chain of α-(1,4) linked galacturonic acid units, with up to 80% of these occurring as methyl esters. Contains up to 4% of rhamnose units, which are then linked to arabinose, galactose and xylose side chains^[1]^.	Thickening agent, suspending agent, stabilizer^[2,5]^, floating beads^[6]^, controlled drug delivery (ocular^[7]^, transdermal^[8]^, colonic^[9,10]^).
Alginate	Linear structure consisting of (1,4) linked β-mannuronic and α-guluronic acids, with proportions depending on the source^[1]^.	Thickening agent, stabilizer^[2,5]^, sustained release agent^[11,12]^, film coatings^[13]^, mucoadhesive systems^[14]^.

2. *Gums containing basic moieties*		
Chitosan	Deacetylated derivative of chitin composed of randomly distributed β-(1–4)-linked glucosamine (deacetylated unit) and N-acetyl-glucosamine (acetylated unit)^[15]^.	Tissue engineering^[16–22]^, wound dressing^[23,24]^ , antibacterial^[25]^, drug delivery^[26]^.

3. *Sulphated gums*		
κ-carrageenan	Disaccharide repeat unit of β-(1,3) linked galactose-4-sulfate and α-(1,4) linked 3,6-anhydrogalactose residues^[1]^.	Thickening agent, gelling agent, stabilizer^[2]^, laxative^[5]^, tablet matrix^[27]^, controlled release agent^[28–30]^.

4. *Gluco and galactomannans*		
Guar gum	Main chain consisting of β-(1,4) mannose units with galactose with α-(1,6) linked branches. Mannose to galactose ratio is 2:1^[1]^.	Binder, disintegrant, thickening agent, emulsifier, laxative^[2,5]^, sustained release agent^[31]^, colon targeted drug delivery^[32]^.
Tara gum	Main chain consisting of β-(1,4) mannose units with galactose with α-(1,6) linked branches. Mannose to galactose ratio is 3:1^[1]^.	Thickener, stabilizer^[2,5]^, controlled release agent^[33–35]^.
Locust bean gum	Main chain consisting of β-(1,4) mannose units with galactose with α-(1,6) linked branches. Mannose to galactose ratio is 4–4.5:1^[1]^.	Thickener, stabilizer^[2,5]^ and controlled release agent (oral, buccal, colonic, ocular and topical)^[36]^.
Konjac	Main chain consisting of β-(1,4) mannose and glucose units with α-(1,3) linked branches. Mannose to glucose ratio is 1.6:1^[1]^.	Gelling agent, thickener, emulsifier, stabilizer^[2]^, Controlled release formulation^[37–40]^.

[1]: Williams and Phillips, 2003a; [2]: Williams and Phillips, 2003b; [3]: Beneke et al., 2009; [4]: Lu, 2003; [5]:Prajapati, 2013; [6]: Sriamornsak et al., 2007; [7]:Giunchedi, 1999; [8]: Musabayane et al., 2003; [9]:Vandamme, 2002; [10]: Wong et al., 2011; [11]: Hodsdon, 1995; [12]: Maiti, 2009; [13]: Rajsharad et al., 2005; [14]: Kesavan et al., 2010; [15]: Islam et al., 2017; [16]: Chung, 2002; [17]: Chung, 2002; [18]: Shalumon, 2009; [19]: Kawakami, 1992; [20]: Hu, 2004; [21]: Wang, 2005; [22]:Mattioli-Belmonte, 1999; [23]: Kumar, 2010; [24]: Madhumathi, 2010; [25]: Rahman Bhuiyan, 2017; [26]:Ali and Ahmed, 2018; [27]:Picker, 1999; [28]: Leong, 2011; [29]: Li, 2014; [30]: Mahdavinia et al., 2015; [31]: Al-Saidan, 2005; [32]: Chourasia and Jain, 2004; [33]: Ma et al., 2017; [34]: Rutz, 2013; [35]:Zeng et al., 2005; [36]: Dionísio and Grenha, 2012; [37]: Alvarez-Mancenido, 2008; [38]: Fan, 2008; [39]: Du, 2006; [40]:Wang, 2014.

#### 2.3.1 Zeta potential measurements

Zeta potential was measured using a Malvern Zetasizer Nano ZS, equipped with an MPT2 auto-titrator (Malvern Panalytical Ltd., Royston, UK). This setup allows the auto-titration and recirculation of sample in an enclosed system with robust and reproducible measurements.

Samples were titrated over a pH range of 2 to 12 using 1 M sodium hydroxide. The titrations were also performed in the reverse direction (i.e., from pH 12 to 2) using 1 M HCl to assess any potential effect of dissolved and dispersed states of the polymers on zeta potential measurements. There were no differences noted in measurement and estimated pK_a_ values. The zeta potential *vs.* pH profiles were then used to determine the maximum zeta potential (Zeta_max_) values for each polymer, i.e., the plateau corresponding to the most-ionised state of the polymer. From the profiles, the pK_a_ value was calculated using pH corresponding to the half of the maximum zeta potential (50% Zeta_max_). All measurements were done in triplicate for each pH and for each polymer concentration, and average estimated pK_a_ ± SD was calculated accordingly.

### 2.4 pH dependant ionisation studies and pK_a_ determination of natural polymers

After method optimisation and validation using commercially available synthetic polymers, a range of natural polymers (Table 2) with widespread use in food and potential pharmaceutical applications were studied to investigate their ionisation behaviour and, when possible, estimate their pK_a_ values. For these polymers, samples were titrated over a pH range of 2–10 to avoid polymer hydrolysis and degradation at extreme alkaline conditions.

## 3 Results and discussion

### 3.1 Zeta potential measurements of synthetic polymers

**Fig. 3:**
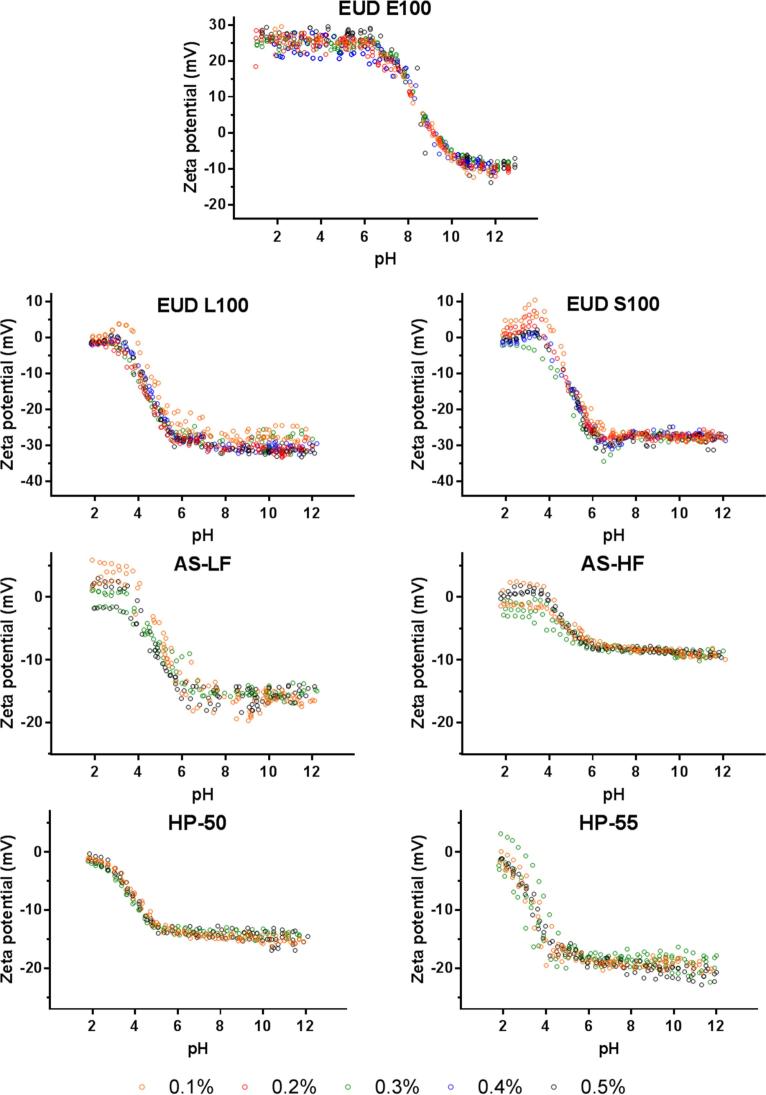
Zeta potential vs. pH profiles of various synthetic polymers at concentrations from 0.1 to 0.5% (w/v) showing no significant effect of changes in concentration on zeta-profiles and pK_a_ estimation.

The zeta potential measurements of the tested synthetic polymers are summarised in Fig. 3, where a clear trend between zeta potential and environmental pH can be seen with all measurements showing an increase in the zeta potential with an increase in the environmental pH. This is not surprising for weakly acidic polymers. However, the opposite was true for EUDRAGIT E100 (a weakly basic polymer) which is more extensively ionised at lower pHs, i.e., pH < pK_a_.

**Fig. 4:**
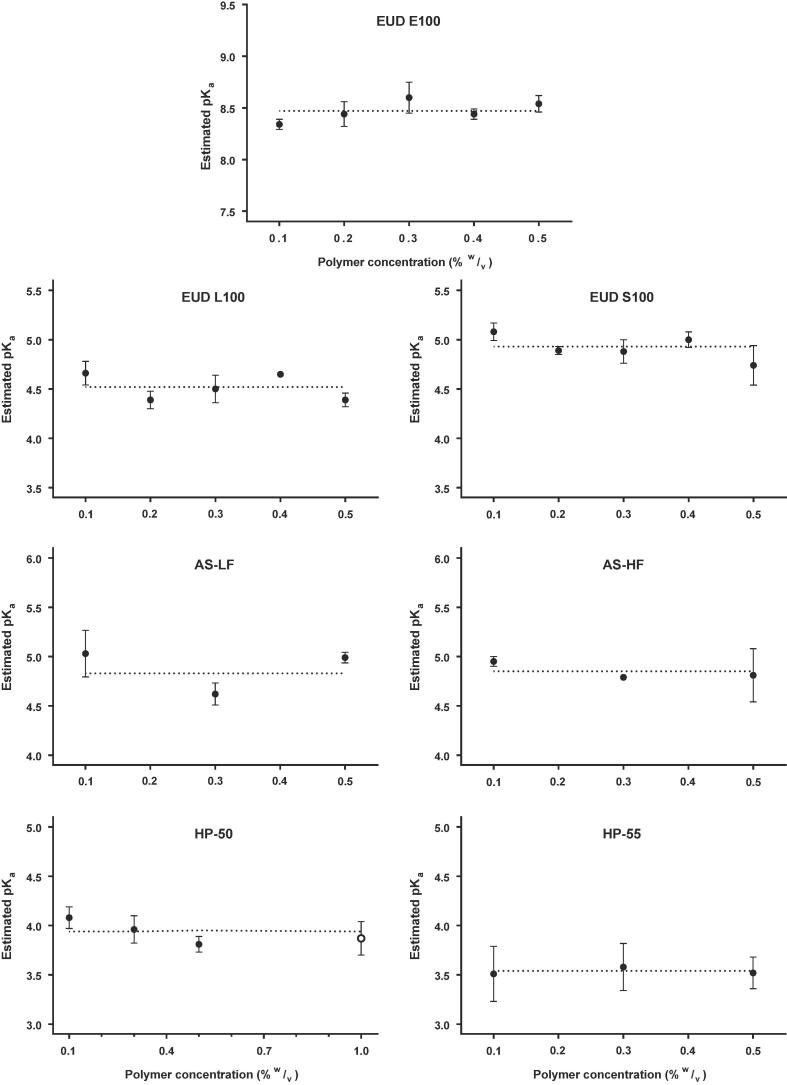
Effect of polymer concentration (0.1–0.5%w/v) on pK_a_ value estimation, where the closed symbols (●) refer the estimated pK_a_ values corresponding to polymer concentration. The open symbol (○) on HP-50 graph represents an additional measurement at 1%w/v polymer concentration to confirm the trend. No significant difference was found between concentrations (p > 0.05).

The weakly acidic polymers exhibited a near-zero zeta potential at low acidic pH (pH ≈ 2), suggesting most of the polymeric species were at their unionised state (>99%) (Fig. 2A). As the pH increases, there is an increase in the ionised fraction (i.e., [HA] to [A^−^]) which results in a net increase in negative charge on the polymer surface causing an increase in the zeta potential which plateaus when most of the HA has been converted to A^-^. Interestingly, the shape of the zeta-profiles was independent of polymer concentrations used (Fig. 3, Fig. 4) and hence increasing the reliability of measured pK_a_ values using this technique.

The Zeta_max_ was determined from zeta profiles and the pK_a_ value of each polymer was estimated accordingly. Table 3 summarises the estimated pK_a_ values using this technique in comparison with the reported literature values.

**Table 3:** Summary of estimated and reported pK_a_ values for the tested polymers.

Polymer	Dissolution pH threshold	Zeta_max_	Estimated pK_a_	Reported pK_a_*
***Synthetic polymers***				
EUDRAGIT E100	≤5.0^[1]^	+24.88 ± 1.66	8.45 ± 0.14	9.0^[2]^
HP-50	≥5.0^[3]^	−14.69 ± 0.89	3.99 ± 0.09	4.20^[4]^
HP-55	≥5.5^[3]^	−19.75 ± 0.95	3.54 ± 0.20	4.49^[4]^; 4.83 ± 0.04^[5]^
HPMC AS-LF	≥5.5^[6]^	−15.25 ± 1.14	4.80 ± 0.20	5.09 ± 0.05^[5]^; 5.10 ± 0.07^[7]^
EUDRAGIT L100	≥6.0^[8]^	−29.88 ± 1.80	4.45 ± 0.13	6.45 ± 0.03^[5]^; 6.62 ± 0.04^[7]^
HPMC AS-HF	≥6.8^[6]^	−8.76 ± 0.29	4.85 ± 0.16	5.15 ± 0.05^[5]^; 4.82 ± 0.03^[7]^
EUDRAGIT S100	≥7.0^[8]^	−27.61 ± 0.59	4.91 ± 0.13	6.66 ± 0.05^[5]^; 6.76 ± 0,03^[7]^

***Natural polymers***				
Gum Arabic		−12.13 ± 0.13	3.20 ± 0.11	3.18 ± 0.02^#^^[9]^
Citrus pectin		−16.05 ± 0.57	3.37 ± 0.04	3.5^[10]^
Alginate		−29.94 ± 1.45	3.45 ± 0.03	3.4^[11]^; 4.4^[12]^
Chitosan		+28.79 ± 1.11	6.75 ± 0.22	6.32 ± 0.02 –6.47 ± 0.03^[13]^

*: potentiometric determinations from literature; ^#^: based on glucuronic acid pK_a_ value in gum Arabic.

[1]: Evonik, 2015; [2]: Quinteros et al., 2011; [3]: Shin-Etsu, 2002; [4]: Davis, 1986; [5]: Riedel and Leopold, 2005; [6]: Shin-Etsu, 2005; [7]: Schmidt-Mende, 2001; [8]: Evonik, 2012; [9]: Fernandes Diniz and Herrington, 1993; [10]: Sila et al., 2009; [11]: Chuang, 2017; [12]: Shinde and Nagarsenker, 2009; [13]: Wang, 2006.

#### 3.1.1 Effect of polymer concentration

It can be argued that a change in polymer concentration may influence the Zeta_max_ and therefore can affect the pK_a_ value estimation. Therefore, the effect of polymer concentrations on pK_a_ value estimation was also studied to ascertain the reliability of the measurement. Interestingly, the concentration of the polymeric dispersion does not seem to affect the pK_a_ value estimation (Fig. 4). In the case of HP-50, however, the estimated pK_a_ value seems to decrease with an increase in polymer concentration from 0.1 to 0.5% w/v. To confirm this behaviour, a higher concentration of 1% w/v was tested and no significant difference (p > 0.05) was found in estimated pK_a_ values across concentrations. Similarly to synthetic polymers, the ionisation profile of the tested gums remained unaffected to the changes in concentrations (data not shown).

From Fig. 2, it can be expected that at an environmental pH two units above the polymer’s pK_a_ value, extensive ionisation would lead to complete dissolution of polymeric chains. However, this may not be the case with every polymer, whilst some may dissolve enough to enable drug release at earlier stages of ionisation, others may only release drug at much later stages.

#### 3.1.2 Hydrophobic effects on zeta potential measurements

Certain polymers (e.g. EUDRAGIT L100 and S100) demonstrate a slightly positive zeta potential at lower pHs (pH 2–4) (Fig. 3), particularly at the lowest concentration studied (0.1% w/v). However, this effect disappears at polymer concentrations ≥ 0.3%w/v. This may be attributed to the non-ionised state of these polymers at low concentrations under acidic conditions.

At low pH (pH ≪ pK_a_), the acidic moieties of the polymeric chains are unionised and undissolved, which increases the polymer’s hydrophobicity compared to when some charged species are present. It has been reported that hydronium ions (H_3_O^+^) behave more hydrophobically than water molecules, accumulating at the interface between water and a hydrophobic media (Vacha, 2008, Luxbacher, 2014). Therefore, at acidic pH, the adsorption of H_3_O^+^ ions to the uncharged polymeric chains creates a slightly positive charged surface at very low polymer concentrations as seen in Fig. 3. On increasing pH, the ionisation of the acidic groups produces a substantially more negatively charged surface and hence an overall negative zeta potential. This effect was absent at higher polymer concentrations (≥0.3%) possibly due to the increased polymer/hydronium ion ratio. The polymeric chains are therefore less densely covered by the positively charged H_3_O^+^ ions. This renders negligible movement of the particles during measurements when a charge was applied during electrophoretic light scattering and generated a signal near 0 mV.

#### 3.1.3 pH dissolution threshold vs. pK_a_

**Fig. 5:**
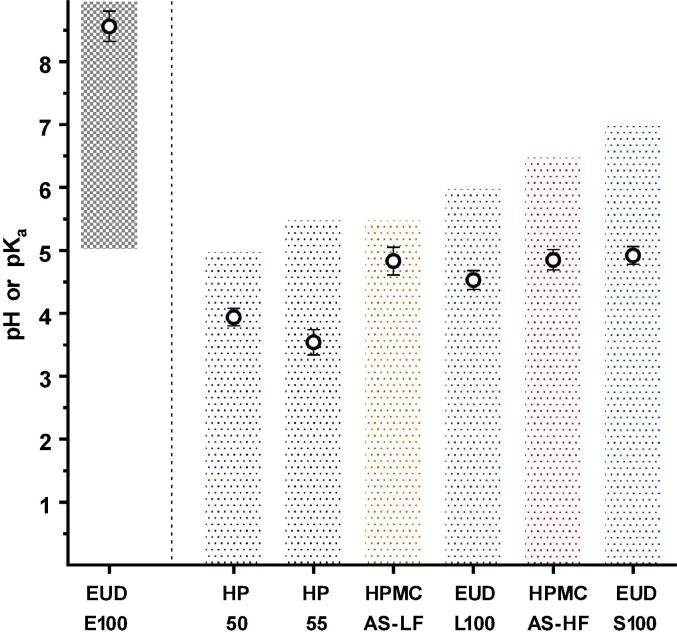
Dissolution behaviour of the tested polymers. The bars represent dissolution pH-thresholds (i.e., shaded areas represent the pH at which the polymers are undissolved). The open circles (○) represent the estimated pK_a_ value (mean ± STD, n = 9), using the proposed technique.

Fig. 5 compares the estimated pK_a_ value of polymers to their reported dissolution pH thresholds. For all enteric polymers except EUDRAGIT E100, it was found that the reported dissolution pH thresholds were always above the estimated pK_a_ value. In contrast, Eudragit E100, a reverse enteric polymer, contains ionisable amine groups. Therefore, complete ionisation (i.e., dissolution) of the polymer is expected below its measured pK_a_ value. As mentioned earlier, the manufacturers do not mention how the dissolution pH thresholds were calculated and there is no known standardisation of approach among different polymer manufacturers. It is likely that some may report complete dissolution of a polymeric film at a given pH while some may rely upon the onset of drug release from the enteric coated dosage form. In our study, the rank order of polymer dissolution pH-thresholds did not follow the measured pK_a_ value for some polymers. For instance, the estimated pK_a_ value for HP-50 was higher than for HP-55 despite its lower dissolution pH threshold. This can be attributed to the polymer structure and the density of acidic (ionisable) moieties on polymer backbone (Table 4).

**Table 4:** Composition of the respective free carboxyl groups of the studied polymers, respective structures and obtained Zeta_max_ values.

Polymers	% ionsable groups	pH Dissolution Threshold	Zeta_max_ (mV)
HP-50^[1]^	21–27% (phthalyl)	5.0	−14.69 ± 0.89
HP-55^[1]^	27–35% (phthalyl)	5.5	−19.75 ± 0.95
HPMC AS-LF^[2]^	14–18% (succinoyl)	5.5	−15.41 ± 1.22
HPMC AS-HF^[2]^	4–8% (succinoyl)	6.8	−8.76 ± 0.29
EUDRAGIT L100^[3]^	46–50% (methacrylic)	6.0	−29.88 ± 1.80
EUDRAGIT S100^[3]^	23–30% (methacrylic)	7.0	−27.73 ± 0.52
			


**A:** Phthalyl group; **B:** Succinoyl groups; **C:** x = Methacrylic acid, y = Methyl Methacrylate. [1]: Shin-Etsu, 2002; [2]:Shin-Etsu, 2005; [3]: Evonik, 2012.

It can be seen from the zeta potential measurements (Fig. 3) that EUDRAGIT L100, HPMC AS-LF and HP-55 have higher Zeta_max_ values compared to their counterparts, EUDRAGIT S100, HPMC AS-HF and HP-50, respectively. This is in agreement with the density of acidic ionisable groups on the polymer (Table 4).

**Fig. 6:**
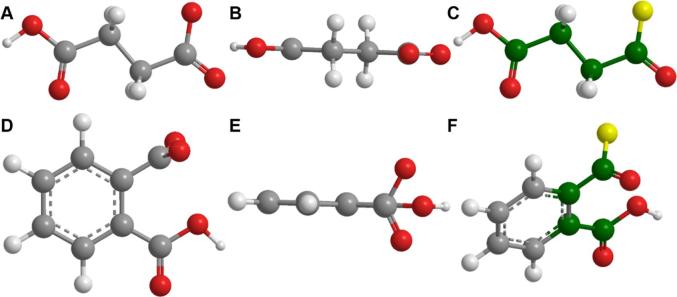
3D structures of succinoyl (A, B and C) and phthalyl (D, E and F) groups. Atoms in green represent rotational bonds. Atoms in yellow represent the binding site to the remaining polymer structure. Figure drawn using information from (Shin-Etsu, 2002, Shin-Etsu, 2005). (For interpretation of the references to colour in this figure legend, the reader is referred to the web version of this article.)

A lower pH dissolution threshold is reported by the manufacturers for polymers containing succinoyl (HPMC-AS) or methacrylic groups (EUDRAGIT S100/L100) corresponding to the higher number of acidic moieties present on the polymer backbone (Table 4). For these polymers, increased density of ionisable species achieves the degree of ionisation needed to show significant dissolution at a lower pH than a polymer with a lower density of ionisable species. The latter would need a higher pH to attain the degree of ionisation needed for the dissolution of the polymeric strands. However, this is not true for the polymers containing a phthalyl group (HP 50/55). In this case, the polymer with higher number of acidic functional groups (HP-55) exhibited the highest dissolution pH threshold. This may be due to the presence of an aromatic acidic moiety that hinders the dissolution of polymeric chains when compared to an aliphatic substituent group (such as HPMC-AS) (Fig. 6). The process of dissolution of a polymer involves water diffusion into the polymer matrix, which eventually leads to the disentanglement of the polymeric chains and consequent dissolution (Fig. 2D). For these polymers, the presence of the aromatic group may influence its solubility by two factors.

Firstly, the aromatic ring creates a more planar spatial conformation (Fig. 6E). Due to a higher number of side-chains on the HP-55 polymer backbone (and thus a higher number of aromatic rings), increased interaction between polymeric chains (π-π interactions and hydrophobic interactions within the aromatic rings) may occur. This may mean more complex entanglement of the polymeric chains, and possibly a slower dissolution. This may explain why the estimated pK_a_ value for HP-50 is higher than the one for HP-55 (3.94 vs. 3.54), even though its dissolution pH threshold is lower. Secondly, the phthalyl group has less conformational flexibility compared to the succinoyl group, as it only contains two rotatable bonds and both are on the same side of the aromatic ring (Fig. 6C) whereas all the carbons in the succinoyl group can freely rotate (Fig. 6F). This causes an increased rigidity in phthalyl groups compared to the succinoyl group leading to less freedom of movement during the disentanglement of the polymeric chains (Fig. 2D). Ultimately, this effect hampers polymer dissolution, despite the ionisation of acidic moieties across polymer chains. Therefore, for these polymers, the presence of aromatic rings possibly plays a more important role in polymer dissolution than its ionisation.

### 3.2 pK_a_ estimation: Zeta potential vs potentiometric determinations

In this work, pK_a_ of various polymers was measured using their ionisation behaviour based on their zeta profiles. The proposed method may present more accurate pK_a_ estimations than the traditional potentiometric determination which is based on measuring bulk solution pH (the concentration of H^+^). However, it is evidenced that the pH at the boundary layer (the interface between the polymeric coatings and the media, Fig. 2D) may greatly differ from the bulk pH (Østergaard, 2014, Taniguchi, 2014, Badawy and Hussain, 2007) and therefore can significantly influence the ionisation and dissolution of these polymers. The boundary layer has an abundance of H^+^ being released from the dissolving polymer which do not diffuse into the bulk solution readily. This renders the boundary layer more acidic than the bulk solution. Potentiometric determinations, therefore, rely on the bulk pH of the media and do not consider the pH within the boundary layer. This leads to an underestimation of the titrant needed to raise the bulk pH thus shifting the titration curve to slightly higher pH values leading to over estimation of pK_a_ values. Therefore, the effective pK_a_ values of these polymers are expected to be lower than the apparent potentiometric determinations.

In contrast, studies involving zeta profiles rely on zeta potential (i.e., charge) determinations using dynamic light scattering. These measurements relate to the net-charge acquired by the dissolving polymer at the boundary layer instead of relying merely on the bulk pH determinations. This leads to lower pK_a_ values estimations than those reported by potentiometric methods (Table 3) and therefore a more accurate representation of ionisation behaviour of these polymeric materials at the boundary layer.

### 3.3 Ionisation and pK_a_ determination of natural polymers

After satisfactory method development and determinations using well-known synthetic polymers, the described method was then employed to study the ionisation behaviour of some commonly used natural gums (polysaccharides) over a range of pHs. The studied polysaccharides differ significantly in their chemical structures and distinctive ionisation behaviour was found from their zeta profiles.

#### 3.3.1 Gums containing acidic moieties

**Fig. 7:**
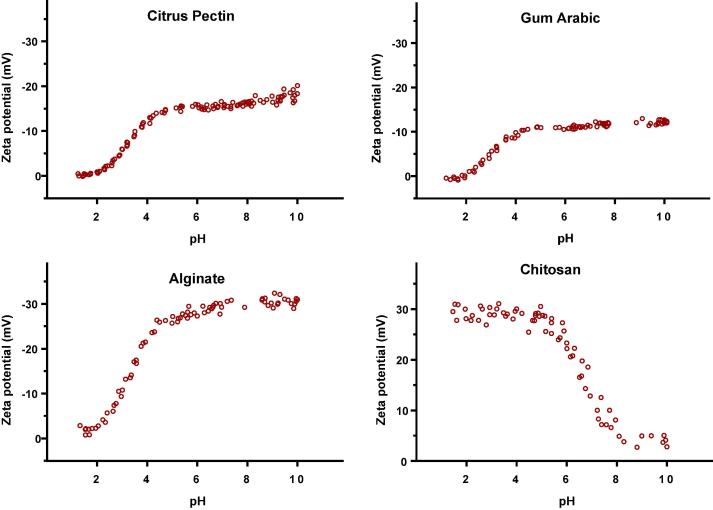
Zeta potential vs. pH profiles of polysaccharides containing acidic (Citrus pectin (0.3% (w/v)), Gum Arabic (0.3% (w/v)) and alginate (0.05% (w/v)) and basic (Chitosan (0.1% (w/v)) moieties.

This group represented gums containing sugar acids. They comprise sugar monomers in which terminal hydroxyl groups are oxidised to carboxylic acids forming uronic acids. The presence of these ionisable groups may therefore play an important role in the polysaccharide dissolution. From this group of polysaccharides, gum Arabic, citrus pectin and sodium alginate were studied and their ionisation behaviour is shown in Fig. 7 and estimated pK_a_ values are summarised in Table 3.

For pectin, gum Arabic and alginate, the shape of the zeta profiles corresponded to the typical weak acid ionisation behaviour as found with gastro-resistant polymers, which can be attributed to the presence of uronic acids moieties in the polymeric structure. Alginate has a much higher Zeta_max_ than gum Arabic and citrus pectin, arising from differences in their polymeric structures. Gum Arabic comprises a chain of galactose units containing acidic units only at the terminus of each branch (Table 2). Citrus pectin contains a long-chain of galacturonic acid units; however, 80% of these are in the form of methyl esters, hence reducing the number of available ionisable groups. Alginate, on the other hand, has a linear structure comprising repeating units of mannuronic and guluronic acids. This explains why a higher Zeta_max_ was found in alginate compared to pectin and gum Arabic. The ionisation behaviour of these polymers was similar to those employed in a typical gastro-resistant formulation. Therefore, these polymers have been extensively investigated in formulating modified-release drug delivery systems (Lu, 2003, Sriamornsak et al., 2007, Vandamme, 2002, Wong et al., 2011, Maiti, 2009, Kesavan et al., 2010, Wang, 2014, Chuang, 2017, Reis, 2006, Lambert et al., 2008, Sansone, 2011, Luo et al., 2015, Arroyo-Maya and McClements, 2015, Alvarez-Lorenzo, 2013, Zhang, 2014, Wu, 2016, Villena, 2015, Shi, 2016, Shi, 2013, de Barros, 2015, Czarnocka and Alhnan, 2015, Chen and Subirade, 2006, Bagheri, 2014, Albertini, 2010).

Probiotic Pearls™ is a commercially available example containing a blend of gelatin and pectin in the outer layer to provide gastric acid protection to encapsulated probiotics (NaturesWay, 2011a, NaturesWay, 2011b). These systems, however, are more suitable for drug delivery to the distal gut, such as the colon than a conventional gastro-resistant application targeted to the proximal small intestine. Nutrateric® is another commercially available coating formulation comprising a pH independent ethylcellulose film containing alginate (Colorcon®, 2015), which acts as a pH dependent pore-former. There are, however, some reports in the literature of premature drug release in gastric conditions and much delayed drug release in small intestinal conditions post-gastric emptying with alginate-based formulations (Czarnocka and Alhnan, 2015, Merchant, 2009).

#### 3.3.2 Gums containing basic moieties

Chitosan was selected to represent gums containing basic moieties and the zeta potential profile of chitosan is shown in Fig. 7. As expected, chitosan shows maximal ionisation at pH ≪ pKa, similarly to EUDRAGIT E100, the commercially available reverse-enteric polymer. At low pH (~2–4) the amine groups in chitosan are fully ionised producing a maximum zeta potential which drops as the pH increases and polymer becomes less ionised. The versatility of chitosan has prompted extensive studies in designing immediate release (Rasool et al., 2012, Imai et al., 2000) and controlled release (Imai et al., 2000, Shi et al., 2008, Yuan, 2010) drug delivery systems.

#### 3.3.3 Gluco and galactomannans

**Fig. 8:**
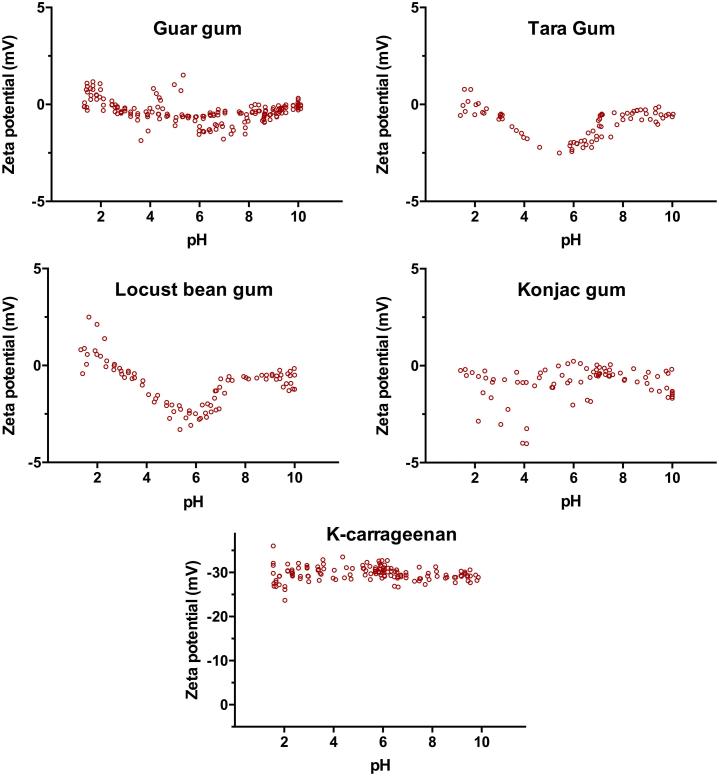
Zeta potential vs. pH profiles of the studied neutral (Guar, Tara, Locust bean and Konjac gums) and sulphated (K-carrageenan) polysaccharides at concentration 0.1% (w/v).

Gluco- and galactomannans are widely used natural gums comprising a mannose backbone with glucose or galactose side chains, respectively. These polymers are mainly composed of the two sugars, which do not contain any ionisable moieties, therefore, are referred to as neutral polysaccharides. From this group of polysaccharides, Guar, Tara, Locust bean and Konjac gums were studied and their zeta profiles are shown in Fig. 8. As expected, all four gums show a zeta potential near zero mV throughout the tested pH range. The absence of acidic or basic (i.e. ionisable) groups causes the gum to maintain neutrality, and therefore a pK_a_ value estimation is not applicable.

#### 3.3.4 Sulphated polysaccharides

Marine algae produce sulphate-containing polysaccharides, such as fucans, ulvans and carrageenans (Patel, 2012). Fig. 8 shows the ionisation behaviour of κ-carrageenan, a sulphate-containing polysaccharide, which attained a highly charged ionised state (Zeta_max_ = −30 mV) over the entire pH range used in this study (pH 2–10). Contrary to the weak acid groups (for instance carboxylic acids) found in other natural gums, these polysaccharides contains sulphate groups. Sulphates are the conjugated base of hydrogen sulphate formed from sulphuric acid, which is a strong acid and dissociates completely in water to form sulphate ions. Carrageenans have been studied for drug delivery purposes, showing promising uses both in immediate release (Ghanam and Kleinebudde, 2011) and in controlled-release formulations (Picker, 1999).

## 4 Conclusion

A novel, simple and inexpensive method for the estimation of the pK_a_ value was successfully developed and employed to study the ionisation behaviour of various synthetic and natural polymers. This method will allow a better understanding of the dissolution behaviour of polymers within the gastrointestinal tract to aid rational design of drug delivery system. The proposed technique will also help in standardising dissolution-pH thresholds across a range of synthetic and natural polymers.

## Declaration of Competing Interest

None.
